# Traumatic Visceral Pneumothorax Resulting from Artificial Pneumothorax

**Published:** 1915-12

**Authors:** Stevens T. Harris

**Affiliations:** Atlanta, Ga.


### Journal-Record of Medicine

Successor to Atlanta Medical and Surgical Journal, Established 1855
and Southern Medical Record, Established 1870

**OWNED BY THE ATLANTA MEDICAL JOURNAL COMPANY**

PUBLISHED MONTHLY

*Offic Organ Fulton County Medical Society, State Examing Board, Atlanta, Birmingham and Atlantic Railroad Surgeon's Association, Chattahoochee Valley Medical and Surgical Association, Etc.*

RICHARD R. DALY, M. D., Editor
BERNARD WOLFF, M. D., Supervising Editor
A. W. STIRLING, M. D., C. M., D. P. H.; J. S. HURT, B. Ph., M.D.
GEO. M. NILES, M. D., W. J. LOVE, M. D., (Ala.); Associate Editors
E. W. ALLEN, Business Manager

**COLLABORATORS**

W. F. WESTMORELAND, M. D., General Surgery
F. W. McRAE, M. D., Abdominal Surgery
ALLEN H. BUNCE, Medical Societies
E. B. BLOCK, M. D., Diseases of the Nervous System
MICHAEL HOKE, M. D., Orthopedic Surgery
CYRUS W. STRICKLER, M. D., Legal Medicine and Medical Legislation
E. C. DAVIS, A. B., M. D., Obstetrics
E. G. JONES, A. B., M. D., Gynecology
ALLEN H. BUNCE, M. D., Pathology and Bacteriology
R. T. DORSEY, JR., B. S., M. D., Medicine
L. M. GAINES, A. B., M. D., Internal Medicine
GEO. C. MIZELL, M. D., Diseases of the Stomach and Intestines
L. B. CLARKE, M. D., Pediatrics
EDGAR PAULLIN, M. D., Opsonic Medicine
THEODORE TOEPEL, M. D., Mechano Therapy
R. R. DALY, M. D., Diseases of the Eye, Ear, Nose and Throat
BERNARD WOLFF, M. D., Diseases of the Skin
E. G. BALLENGER, M. D., Diseases of the Genito-Urinary Organs

VOL. LXII. ATLANTA, GA., DECEMBER, 1915. NO. 9

### TRAUMATIC VISCERAL PNEUMOTHORAX RESULTING FROM ARTIFICIAL PNEUMOTHORAX.

BY STEVENS T. HARRIS, M. D., ATLANTA, GA.

The term PNEUMOTHORAX, while no doubt explanatory of a condition present, does not comprehend the varied etiology of the condition described. In order that this may be corrected, various modifying words have been used in conjunction, as

ARTIFICIAL PNEUMOTHORAX, specifying one intentionally artificially produced; PATHOGENIC PNEUMOTHORAX, one the result of disease; TRAUMATIC PNEUMOTHORAX, following injury or unintentional surgical procedure; SPONTANEOUS PKEUMOTHORAX, occurring from no known cause.

Theer is still another type which occurs following ARTIFICIAL PNEUMOTHORAX where there is more or less trauma resulting from the production of the pneumothorax—the break occurring through tne visceral pleura at a site which is or has been diseased. Indeed we might still further modify the nomenclature by PARIETAL, VISCERAL or DIAPHRAGMATIC, indicating from which direction the air or gas is introduced.

During or following the production of ARTIFICIAL PNEUMOTHORAX we occasionally have a break through the viscera communicating with a bronchus, as above described. It is this type which I wish to discuss.

The following cases will illustrate the three phases which we have observed.

Case 1. Miss C., age 22. Florid type with most active tuberculous lesions in right lung. Severe intoxication, chills and high temperatures. Of about eight or ten weeks standing. She had been treated for both malaria and typhoid fever during this time. Successful pneumothorax was done on right side, followed by very rapid disappearance of all symptoms of tuberculosis. In six weeks, without forced feeding or open air treatment and despite the heat of July and August in South Georgia, she gained so much weight that clothes used previous to her illness had to be altered. At the end of this time circumstances forced us to relinquish the case and she was sent to Atlanta, Ga., to Dr. H. F. Harris for a continuation of the pneumothorax. Miss C. was most earnestly admonished that the pneumothorax must be kept up for not less than a year.

In two or three months she and her family considering it no longer necessary to continue the treatment, discontinued it. For about six months she appeared to be in perfect health, engaging in social affairs, outdoor sports, horseback riding, etc.

At this time a severe attack of grippe and pleurisy in the right side occurred. Shortly she again came under our care, being most desirous of a reproduction of the pneumothorax, the left side being more involved than at first and the right side again active.

On account of adhesions resulting from the pleurisy, numerous attempts were made before a partially successful pneumothorax was accomplished on the right side. In spite of fairly high pressures the lung was held out in several places by strong adhesions. The beneficial influence of a partial pneumothorax soon began to manifest itself and the patient made satisfactory progress.

Finally, in an effort to break down the adhesions by very high pressure, a visceral break was caused. Infection of the pleura quickly followed, resulting in a pyo-pneumothorax of the worst kind with its train of evil consequences.

With the patient in certain positions, a filling of gas would cause the most profuse discharge of foul pus through the bronchial tubes. A filling of gas ending with high pressure would in a very short time sink to a much lower pressure, showing its escape. A drainage established through the thoracic wall did not deter a fairly rapid fatal termination.

Case 2.—Mr. O., young man. Tuberculous infection in both lungs. Doing fairly well under open air, etc., treatment. Developped uncontrollable hemorrhage from left lung. As a last resort artificial pneumothorax was applied to the left side, only about 250 c. c. of gas being introduced. The hemorrhage was promptly controlled. The next day 200 or 300 c. c. more gas were added as a precaution. At the end of the filling the intra-pleural pressure was strongly negative. In a few hours deep and subcutaneous emphysema manifested itself and rapidly extended to trunk, neck, head and limbs. The introduction of a needle into the pleural cavity gave manometric reading of +3 to +6 of c. c. water pressure, thus proving a visceral break. Withdrawal of gas only temporarily reduced the pressure. A silver tube with an outside filter was introduced between the ribs into the pleural cavity in an effort to reduce the intrapleural pressure, hoping thereby to stop the progress of the emphysema. The emphysema was caused by air which was inspired into the pleural cavity through the break in the lung, and when expiration began the increasing intra-pleural pressure closed the break in the lung with a valve effect and the air was forced out through the opening in the parietal pleura at the site of the needle puncture. The introduction of the silver tube seemed to modify the condition somewhat and if more thoroughly done, might have resulted in a stoppage of the emphysema.

The fatal termination of this case appeared to be due to the embarrassment of vital organs by the deep emphysema and likely a general sepsis spread throughout the body by the contaminated air.

Case III.—Mr. G , age 60. Long standing case. Tuberculous infection more or less throughout both lungs, with multiple cavities. With recent most rapid decline of patient.

Over our protest patient and member of family insisted upon pneumothorax and assumed full responsibility. The outlook was utterly hopeless in any event. The left lung being selected as the worse, on a second attempt about 350 c. c. gas was introduced and a pneumothorax established without disturbance to the patient. At the end of the filling there was a strong negative pressure and there had been indications of many adhesions. In about 30 minutes sudden distress was manifested, with much shock, severe dyspnoea, cyanosis, and rapid and feeble heart action. Immediately after the attack began a needle was introduced into the pleural cavity and manometric readings similar to those in Case 2, were registered. Several hundred c. c. of air was aspirated into the pneumothorax instrument from the pleural cavity with only a momentary reduction of the intra-pleural pressure.

The condition of the patient remained practically the same for about 17 hours, at the end of which time he, refusing to lie down and while sitting in a chair perfectly conscious, toppled over dead.

While the embarrassment to respiration in this case was most marked, it seemed quite clear that the circulatory system was the first to give way. It is easy to understand how the sudden displacement of the heart and mediastinum to the right by a sudden massive pneumothorax in the left side would cause the most intense embarrassment to the circulatory system, especially if we add to this the daming back of the circulation from a suddenly collapsed and compressed lung.

Further factors in this condition, as described in the last case, would be the imperfect circulation in the diseased lung opposite the pneumothorax; the heart already weakened from chronic toxic myo-carditis; the oxygen starvation and carbon dioxid poisoning of the musculature and higher centers.

In the light of the technic of artificial pneumothorax as perfected up to the present time, the condition described in the above cases constitute the chief dangers in its application. But, fortunately, such mishaps are comparatively rare and should not in the least be considered prohibitive.

The lessons and conclusions drawn from the conditions noted would be first,—that it is very apt to prove fatal: second,—that very high pressure should not be used especially for the purpose of breaking down adhesions: third,—it is impossible to foresee the occurrence of this condition in any given case, although we might surmise the possibility of it in cases with many adhesions and cavities near the periphery: fourth, —the condition of the opposite lung should be sufficiently good for the proper aeration of the blood: fifth—the circulatory system should be thoroughly investigated before operation: sixth —after the visceral break occurs likely the best way to combat the increased pressure and emphysema which might occur would be by an equalization of the intra-pleural pressure with the outside atmospheric pressure by creating a more or less permanent opening through the thoracic wall: seventh—a pyopneumothorax occurring in this condition is apparently not benefited by drainage: eighth—the direct cause of a fatal termination in this condition might be pyo-pneumothorax, the results of emphysema, or the results of mechanical displacements or mechanical interference with the circulatory system: ninth—a pneumothorax once established should under only exceptional cases be allowed to completel disappear until the process has been carried to its logical conclusion.

719 Hurt Building.

